# How Leaders at High-Performing Healthcare Organizations Think About Organizational Professionalism

**DOI:** 10.1017/jme.2024.174

**Published:** 2024

**Authors:** Julie L. Agris, Sherril Gelmon, Matthew K. Wynia, Blair Buder, Krista J. Emma, Ahmed Alasmar, Richard Frankel

**Affiliations:** 1. SHEPARD BROAD COLLEGE OF LAW, NOVA SOUTHEASTERN UNIVERSITY, FORT LAUDERDALE, FLORIDA, USA; 2. OHSU-PSU SCHOOL OF PUBLIC HEALTH, PORTLAND, OREGON, USA; 3. UNIVERSITY OF COLORADO, CENTER FOR BIOETHICS AND HUMANITIES, AURORA, COLORADO, USA; 4. DEPARTMENT OF INTERNAL MEDICINE, UNIVERSITY OF COLORADO SCHOOL OF MEDICINE, AURORA, COLORADO, USA; 5. DEPARTMENT OF HEALTH SYSTEMS MANAGEMENT & POLICY, COLORADO SCHOOL OF PUBLIC HEALTH, AURORA, COLORADO, USA; 6. STONY BROOK UNIVERSITY, OFFICE OF EDUCATIONAL EFFECTIVENESS, STONY BROOK, NEW YORK, USA; 7. INDIANA UNIVERSITY SCHOOL OF MEDICINE, INDIANAPOLIS, INDIANA, USA

**Keywords:** Organizational Professionalism, Performance Improvement, Patient Partnerships, Community Partnerships, Organizational Culture

## Abstract

This pilot study is the first formal exploration of the concept of “Organizational Professionalism” (OP) among health system leaders in high-performing healthcare organizations. Semi-structured key informant interviews with 23 leaders from 8 healthcare organizations that were recipients of the Malcolm Baldrige National Quality Award (MBNQA) or Baldrige-based state quality award programs explored conceptualization, operationalization, and measurement of OP. Further exploration and understanding of OP in healthcare organizations has the potential to establish and sustain professional and ethical organizational cultures that bolster trust through the sound implementation of laws, policies, and procedures to support the delivery of high-quality patient care.

## Introduction

Professionalism in health services delivery typically describes the rights, duties, and obligations of certified *individuals* who offer their services to the public. But recent scholarship has expanded the scope of inquiry to include organizations and their rights, duties, and obligations, invoking the concept of “organizational professionalism” (OP).^1^ One line of research, for example, has shown that an organizational framework that embodies a “Culture of Professionalism” is critical to healthcare providers’ well-being and their ability to provide excellent patient care and support public trust in healthcare entities.^2^

There is not yet a single, widely accepted definition of OP. A better understanding of the definition, implementation, and measurement of OP in healthcare organizations may facilitate the creation and long-term sustenance of desirable organizational cultures in the health sector. With an improved understanding of this concept, healthcare organizations that understand, measure and seek to improve their OP may develop knowledge and strategies to intentionally implement laws, policies, processes, and procedures informed by OP principles. In turn, a more robust development of OP in healthcare organizations may support leadership and modeling for sustaining organizational cultures that bolster trust and support the efforts of individual healthcare professionals as they strive to deliver healthcare services to their patients in a professional, ethical manner.

The “Charter on Professionalism for Health Care Organizations” (the Charter) defined OP with reference to four domains that encompass the organizational competencies necessary to sustain and enhance a “Culture of Professionalism”: (1) patient partnerships, (2) organizational culture, (3) community partnerships, and (4) fair business practices. These domains match concepts in professionalism which articulate that trust in professionals arises when they meet expectations in relations with patients, colleagues, community, and business associates. The application of these concepts to foster trust in organizations has intuitive appeal, but the Charter was developed by leaders in academic medicine, and it is not known whether its concepts are useful to leaders in other settings.

The “Charter on Professionalism for Health Care Organizations” (the Charter) defined OP with reference to four domains that encompass the organizational competencies necessary to sustain and enhance a “Culture of Professionalism”: (1) patient partnerships, (2) organizational culture, (3) community partnerships, and (4) fair business practices. These domains match concepts in professionalism which articulate that trust in professionals arises when they meet expectations in relations with patients, colleagues, community, and business associates.

The research study described here sought to understand whether and how leaders in high-performing healthcare organizations understand OP, how they might measure performance in its four domains, and if and how they might use the concepts in the Charter and its associated domains to build trust in their organization.

To explore the Charter’s practical applications, the authors first engaged a panel of ten experts, including individuals with clinical and academic backgrounds in medicine, health services, law, public health, organizational behavior, communication, and ethics who have contributed to the exploration and literature on professionalism in medicine. The panel considered the Charter, including its implementation (or lack thereof) and the lack of related measures and metrics, and recommended conducting a qualitative study with leaders of high-performing healthcare organizations to better understand their knowledge and use of the concept of OP. We prepared a summary of the positions/roles of healthcare leaders interviewed while also protecting the anonymity of interviewees (**Table 1)**.

**Table 1:** Positions/Roles of Interviewees

Leadership Position/Role	Number of Interviewees	% of Interviewees
Executive/Operations/Strategy	8	35
Medical/Nursing/Other Clinical	7	30
Quality/Safety/Performance Improvement/Patient Experience	8	35
**Total**	**23**	**100**

Since the proposed definition of OP needed to be reality-tested among organizational leaders, and there are no widely accepted metrics for assessing OP and therefore no resource for identifying high-performing organizations regarding OP, we elected to gather experiences of executives using in-depth qualitative interviews. We chose to interview leaders at organizations widely considered as high performers in health services delivery. For this purpose, we selected healthcare organizations that were recent recipients of the Malcolm Baldrige National Quality Award (Baldrige Award), which we considered as a proxy for a high likelihood of having embraced performance in regard to OP or OP-like themes. Since the language used in the Charter is not identical to terminology used in the Baldrige standards, we created a crosswalk of language used in the Baldrige “Core Values and Concepts” and corresponding OP domains (**Table 2**), which we used in developing our interview guide.

**Table 2:** Baldrige Language to Organizational Professionalism Language Crosswalk

**Baldrige** **Core Values and Concepts**	Organizational Professionalism Domain(s) Containing the Baldridge Concept
Systems Perspective	Organizational Culture
Visionary Leadership	Organizational Culture
Customer-Focused Excellence	Patient Partnerships
Valuing People	Patient Partnerships Organizational Culture Community Partnerships
Agility and Resilience	Organizational Culture
Organizational Learning	Organizational Culture
Focus on Success and Innovation	Organizational Culture
Management by Fact	Organizational Culture Community Partnerships Operations and Business Practices
Societal Contributions	Community Partnerships
Ethics and Transparency	Organizational Culture Operations and Business Practices
Delivering Value and Results	Organizational Culture Operations and Business Practices

**Note:** (NIST, n.d.(b) and Egener et al., 2017).

The Baldrige Award was used as the criterion to select healthcare organizations for this project as it is the highest level of national recognition for performance excellence that a US organization can receive.^3^ It was established by the US Congress in 1987 to recognize and improve performance of the nation’s businesses, hospitals, schools, nonprofit organizations, and government agencies.^4^ The Baldrige Program recipients have contributed to more than $29 billion in economic benefit and the ratio of benefits for the US economy to Baldrige Program costs is 820:1.^5^ The healthcare sector award was initiated in 1999^6^ and 28 healthcare organizations have received the Baldrige Award since 2002.^7^ State, local, and regional Baldrige-based award programs use the Baldridge Excellence Framework to advance organizational excellence and competitiveness in states and regions. These programs help many local organizations start and continue their performance excellence journeys.

## Methods

After initial consultation with Baldrige program leadership, we identified healthcare organizations that had received the national Baldrige Award between 2018 and 2022. Ten of these organizations were purposively selected to achieve a sample with diversity in geographic location, ownership, academic affiliation, faith-based or secular, urban/rural, organizational structure, and size; leaders from six of these organizations agreed to participate. Four organizations were unable to participate due to limited resources available at the time of invitation and the ongoing effects of the COVID-19 pandemic. To supplement this sample, we invited selected award recipients of state Baldrige-based programs which yielded two additional organizations as participants. Each participating organization was invited to identify two or three senior leaders who had broad perspectives on the organization; one of the senior leaders interviewed at each organization was their lead for the Baldrige application. Study protocols were approved by the Stony Brook University Institutional Review Board (IRB #IRB2021-00167).

We developed a semi-structured interview guide, organized around the OP domains as modified to reflect Baldridge terminology **(Table 2)**. The interviews included three main sections: (1) exploring the interviewee’s familiarity with the concept of OP; (2) describing how the organization approaches each of the proposed domains of OP, focusing on measurement strategies; and (3) exploring additional possible domains that these executives thought could be related to OP. In the second section, for each domain we first asked how the organization worked to ensure high performance in the domain, and then we asked in what ways, if any, the organization measured its performance in that domain. The third section comprised an open discussion of any additional domains that the interviewee saw as conceptually related to OP. We pilot tested the interview guide with four healthcare leaders not included in the study and made minor revisions to ensure understandability of the questions. Subsequently, pairs of researchers from our team conducted virtual, individual, semi-structured key informant interviews with twenty-three leaders from the eight selected healthcare organizations. Each interviewee signed a consent form and agreed to have their interview recorded.

The interviews were transcribed verbatim, checked for accuracy, and all personal identifiers were removed before they were uploaded to Atlas.ti™^8^ for analysis. Interviews were then analyzed using immersion/crystallization to identify provisional themes.^9^ Iterative consensus-building among multiple reviewers and double coding of transcripts were used to identify emergent themes, resolve differences, and agree upon final coding categories.

Initial coding guidelines and protocols were developed prior to coding, utilizing a constructivist grounded theory approach.^10^ The initial codebook was developed through open coding with preliminary codes and subcodes, using one transcribed interview. An additional nine interviews were coded to refine the initial codebook, to reflect consistent and accurate representations of themes across interviews. The refined codebook was inputted into Atlas.ti™ where the remaining interviews were subsequently coded by one of three coders, who had regular discussions to resolve/reconcile any inconsistencies, make necessary modifications to the coding categories in the codebook, and discuss emerging themes of particular importance. Iterative consensus-building among the three coders facilitated agreement on domains and coding categories, and the codebook was modified throughout the data analysis process to reflect this consensus, based upon iterative comparisons between the previously coded and newly analyzed data.^11^

Upon completion of initial coding, the coded interviews were merged into one combined project file in the Atlas.ti™ program. From this merged file, each coder was assigned different interviews to double-code to optimize consistency and reliability of results. Following double-coding, the three coders jointly reconciled any noted discrepancies, reviewed/clarified any changes to the codebook, and compared reflections. They also identified primary themes that emerged, based upon the coding results, such as factors that were consistently cited or noted as relevant to OP by the interviewees. These themes were then utilized for interpretation of findings.

## Results

Several themes arose across all three sections of the interviews, such as the importance of building and sustaining patient and community trust, but in general each proposed domain of OP elicited a set of unique themes. We present these below in the order in which they were discussed in the interviews.

### Healthcare Leaders’ Conceptualization of OP

We anticipated that some leaders from Baldrige recipient organizations might be unfamiliar with the concepts and domains of OP. Therefore, the first part of the interview explored OP and allowed the interviewee to provide their own definition of OP.

Few interviewees had read or heard about the specific concept of OP prior to our interviews, yet all rapidly recognized connections between OP principles and their organization’s activities, performance metrics, and goals. It was also notable how easily most interviewees adopted the language of OP and used this throughout the interviews. Several described the potential for OP to help frame a more expansive and useful understanding of professionalism for themselves and their organizations. For example, one interviewee stated:

…previously, I thought [of] professionalism as our image, or maybe even certifications or education. But just thinking… some of the questions that you’re asking about, some of our partnerships, and the community influence…and I think, really, what I’m zeroing in on is how… are we specifically measuring some of these things, and are we able to improve?

(Quality/Safety/Performance Improvement/Patient Experience Interviewee)

All interviewees also recognized relationships between OP principles and their organization’s core values. Quotations related to this finding are presented in **Table 3** as “Acknowledgment of OP Principles Shifts Thinking About OP.”

**Table 3:** Sample Quotations Representing Charter Domains, Additional Emerging Themes, and Additional Observations from Healthcare Organizations

Category	#	Identified Example/Quote
*Charter Domains*		
Patient Partnerships	PP1	“One of the things we do is: we have a patient priority, which isn’t a medical or nursing diagnosis, but it’s something that helps them feel comfortable and make sure that their voice is heard while they’re here in the hospital. So, it may be something like, ‘I want to make sure that my blinds are open every morning and closed every night, because I do that at home and I can’t do that here.’ Or, ‘I want to call my grandson or granddaughter every night, because I talk to him every night, and I don’t necessarily have the means to do that independently here in the hospital.’ And so, that way, we’re listening to what they have to say.” (Quality/Safety/Performance Improvement/Patient Experience Interviewee).
	PP2	“So, how a patient or family are approached and treated, from the point of entry onto our campus, all the way through the time they leave reflects the professionalism of our organization. And that is reflected in each and every interaction they have, whether it’s with the parking attendant or passing the CEO in the hallway.” (Quality/Safety/Performance Improvement/Patient Experience Interviewee).
	PP3	“I think, previously, I thought [of] professionalism as our image, or maybe even certifications or education. But just thinking… some of the questions that you’re asking about some of our partnerships, and the community influence, and I think, really, what I’m zeroing in on is how…are we specifically measuring some of these things, and are we able to improve? That piece of it is really where I’m focusing. Maybe we have approaches to do this, and we have processes in place, but how are we measuring effectiveness? So, I think that that’s something I’ll take and run with.” (Quality/Safety/Performance Improvement/Patient Experience Interviewee).
Organizational Culture	OC1	“We [have] 2,600 employees. We show up in the morning with our hearts open, and we walk in relationship with each other and with community. We’re responsible for that concept of shared responsibility, relationship, and noticing and valuing dignity in the way that we’re all walking every day.” (Executive/Operations/Strategy Interviewee).
	OC2	“We talk about being the best, and that’s a broad thing to say: ‘We want to be the best.’ And what that means to us is: we want to continuously improve. We know that being the best isn’t really a destination because we constantly need to improve. …We need to stay on that journey. We make sure that we’re not telling people, ‘We want to be the best, and if you’re not the best, then you can’t be part of our team.’ But rather, ‘We want you to be open to feedback. We want you to provide us feedback if you see an opportunity for us to improve. And we’re on this journey together. We want to help you be the best version of yourself. And we want you to help us be the best version of ourselves.’ That’s the opening line when people are interviewing, as well as when they start here. And then, we make sure that we align the orientation process with that.” (Quality/Safety/Performance Improvement/Patient Experience Interviewee).
	OC3	“You really have to have that culture from an organizational standpoint. And that culture definitely is set by senior leadership. But really, that has to be infused at all levels. And I think that, when you have a culture that supports that professionalism as an organization, it doesn’t matter if your role is the CEO or you’re the courier between clinics. And I think that you have to be very intense with that. No matter what our role is, we all have ‘always behaviors.’ … It’s our expectations. And one of the things that I am actually very proud of is, about 11 years ago, we basically did a grassroots of redefining what we called our ‘always behaviors’ with frontline associates. And obviously, this was endorsed by our administration. But really it helped us to set a tone at all levels, to say that ‘We are accountable to each other.’” (Medical/Nursing/Other Clinical Interviewee).
	OC4	“It’s intentional that our senior leaders are at every orientation, every two weeks, Monday morning, 8 a.m. They are physically there as a team. And I think that, when you talk about vision intentionalism, that’s not something that we just leave by chance. That’s an intentional move that, as an associate comes on, they understand [that] this is our culture. Here is the professionalism that’s being displayed, even by your senior leadership.” (Medical/Nursing/Other Clinical Interviewee).
	OC5	“Lack of accountability, to me, is probably the number one eroder of professionalism in organizations, especially in healthcare.” (Quality/Safety/Performance Improvement/Patient Experience Interviewee).
	OC6	“We sit in relationship with others through the power of story. And then we’re taught to hide our own story, but expect others to share their nitty gritty with us. How do we show up as real humans with each other, as co-workers, and with customer owners. That’s fundamental to how we want to behave as an organization.” (Executive Operations/Strategy Interviewee).
	OC7	“We get at people’s fundamental needs, both as a patient and as an individual working for us. I think the professionalism aspect of that is to create urgency to get it done. Because when you look at how organizations work, many times, they’re slow. They’re bureaucratic or they don’t take risks.” (Executive Operations/Strategy Interviewee).
	OC8	“Ultimately, it’s senior leaders. We know … [culture] rises or falls on frontline management. Because we can talk all day long. I think it starts there, but then, very quickly, it’s getting to those frontline managers to make sure we’re aligned with them, and make sure it’s just not rhetoric at the top. So, I think it starts at the top, but then it flows down through the organization.” (Executive Operations/Strategy Interviewee).
	OC9	“I think the question is: how do you look at that? Is it professionalism? Is a professional, who is maintaining the status quo of a hospital…is that the same as looking at it more holistically? …Somebody could say, ‘I’m incredibly professional because I’m doing everything traditionally like we’ve always done it, and so I’m doing that in the highest, professional manner.’ Or, does professionalism suggest a growth mindset? Does it suggest looking at things differently and more broadly?” (Executive Operations/Strategy Interviewee).
	OC10	“I think of it as a collective culture of professional behaviors, skills, and attitudes that are really exemplified as a whole, that becomes part of the organization’s brand.” (Quality/Safety/Performance Improvement/Patient Experience Interviewee).
	OC11	“We needed a lot of focus on the infrastructure of the organization, and the reason why is because you can have a wonderful thing because of a wonderful leader, or you can build it into the fabric of the organization. And we’ve done a lot of work to build this culture into the fabric of the organization so that it lasts past any one leader.” (Executive Operations/Strategy Interviewee).
Community Partnerships	CP1	“We actually have taken a shift as an organization over the last few years. As an organization, we’re very mission-driven, but, in order to really ensure that we are engaged with our community, we have had a shift. Strategically, we have a senior leader over mission development ensuring that, as we’re moving forward as an organization, we are meeting patients where they’re at.” (Medical/Nursing/Other Clinical Interviewee).
	CP2	“One of our strategic priorities is partner relationships, and that’s with key stakeholders, our community, and it really does get to those relationships with [our] key partners. This one’s a little more challenging to measure and to report, because it’s not like we can check a box, or it’s not like there is a survey that we send to our partners. But, it definitely comes across in other ways. So, we consider our relationship with the city and our relationship with other medical providers outside of our own medical group. Again, I don’t know how one measures that, entirely. But for us-- like our relationship with our physicians who aren’t part of our organization — we measure that through our workforce engagement and physician engagement survey.” (Quality/Safety/Performance Improvement/Patient Experience Interviewee).
	CP3	“We track social media presence; the number of hits. We track our grievance process, in terms of when people, frankly, have a problem with our organizational professionalism. We track positive comments. We hold quarterly PFAC (Patient and Family Advisory Councils) meetings across three work systems. We undertake stakeholder analysis, where we’re seeking feedback from the outside, whether it’s independent physicians, managed care, a whole litany of outside stakeholders, asking them two questions: ‘What does [Organization Name] do well?’ and ‘What can we improve on?’ and how much those parties want to engage with us.” (Executive Operations/Strategy Interviewee).
*Charter Domains*		
Community Partnerships	CP5	“I think it’s accountability, but I think it’s being a partner. We’re trying to become a better partner with community members, whether that’s a Boys and Girls Club, or a homeless shelter, or a food bank.” (Medical/Nursing/Other Clinical Interviewee).
	CP6	“We want to make sure that we are the provider of choice, the hospital of choice for our community. But, we also measure, the same type of information through our associates in the annual associate engagement survey, and also our physicians. So, as a place to practice, if our physicians don’t trust us… if they don’t trust everyone from our senior leaders, all the way to the OR team [it’s a problem].” (Medical/Nursing/Other Clinical Interviewee).
	CP7	“We track our volunteerism of our employees. We allow them to sign up to support different events, and then we pay them to participate in those events. We track how many volunteers we’ve had by department, and then organizationally. So, that’s the way that we track community involvement. And then, any financial support that we offer to our community, and we track that through our foundation as well.” (Quality/Safety/Performance Improvement/Patient Experience Interviewee).
	CP8	“We are definitely building strong relationships with our community based on the transparency of the messaging that we put out to the community. So, we do a number of different hosted events and local events that involve the community, and give them a chance to see what it’s like to be a patient at [Organization Name] through storytelling from a prior patient.” (Quality/Safety/Performance Improvement/Patient Experience Interviewee).
	CP9	“There’s something about psychological safety that feeds into a culture of professionalism. So, whether or not our workforce is empowered to speak up at any level would give us a pulse check of professionalism.” (Quality/Safety/Performance Improvement/Patient Experience Interviewee).
	CP10	“The words that I’m using are really challenging the traditional hierarchy in healthcare, where sometimes as healthcare professionals, we see ourselves as experts. We think that we know what’s best about how healthcare should be delivered. And, in our minds, we want to break that hierarchy, and instead realize the idea of a partnership where, as healthcare systems… healthcare professionals, we bring our expertise to the table. So, we value what we bring, and equally value what community brings and try to put those things together on the table. We do that work by thinking about how we sit in partnership at a macro-level between community and system, and at a micro-level between care teams and families.” (Executive Operations/Strategy Interviewee).
	CP11	“We find that [in] healthcare, our data systems are designed to help us bill. And that’s not population health. So, we’ve actually created what we call a ‘data mall’ [that] pulls all that data into one place, so that we have longitudinal data for a specific population of [Racial/Ethnic Group] people, generation to generation. So, we have this really unique way of seeing the impact on community.” (Executive Operations/Strategy Interviewee).
	CP12	“To recognize that we bring expertise, but to be humble enough to know that community does, too, and that they’re going to drive what we should do.” (Executive Operations/Strategy Interviewee).
	CP13	“We have challenged ourselves with a bunch of metrics above and beyond that, from a social determinants of health perspective [on] how we’re trying to improve the community. We do a ton of economic development in the community, and not for financial gain. We’re putting a lot of resources in the community just to improve the health and wellbeing of the community itself. We’re spending those dollars wisely and for the benefit of the community versus a for-profit mentality, where we’re just trying to make more money and grow for the sake of making more money.” (Executive Operations/Strategy Interviewee).
	
Operations and Business Practices	OB1	“We measure ourselves as an employer in the neighborhood. So, how much business do we generate to our neighborhood? We’re the largest employer in our little section of downtown [Geographic Location]. So, obviously, the business practices that we employ for our own staff have a huge impact on this community. We look at the… revenue growth that we bring to the region by being positioned here. And we measure that. That’s a major study that we undertake to quantify ourselves as a good business partner. We do have a very specific initiative around our local suppliers and ensuring that a percentage of our revenue that we spend on supplies actually stays in our neighborhood and benefits the local businesses here. So, we measure that. We also measure our effect on the overall ecology around us. So, we measure our ‘green waste.’ We look at: ‘How do we minimize our carbon footprint?’ And we do all of that measurement around being a good business partner.” (Executive Operations/Strategy Interviewee).
	OB2	“My ongoing lament is that healthcare leaders, especially in not-for-profit healthcare, have really lost their way. And they’ve been lost for decades because they’re so focused on the bottom line. And none of them will admit that they’re so focused on the bottom line. But the flip side of that coin is: they’re just not transforming, and no one is going to the heart of the problem. They’re sitting back. So, we use the Baldrige criteria to help us move faster towards that vision.” (Executive Operations/Strategy Interviewee).
	OB3	“As far as organizational professionalism, I think we’ve grown and learned to recognize rather late (compared to some businesses out there) that you have to have really granular data to be able to make significant changes. It’s easier being good, but going that last 10% and achieving excellence is really challenging. And without accountability and using the data, measuring it, being responsible for those outcomes… And in some cases, it’s tough love. And if you’re not going to be on the bus, then get off. I think we’ve worked really hard at trying to have a very clear strategy, and then have that strategy cascaded down through the front lines using dashboards and other tools to help hold people accountable, as well as transparently communicate out what those expectations and performance milestones are.” (Medical/Nursing/Other Clinical Interviewee).
	OB4	“So, when we talked about the ‘always behaviors,’…we hold each other accountable. It helped us to start having the tougher conversations. And if you have an engaged workforce. So, you can have two nurses. They both come in each day. It’s very easy to tell which one is engaged; is working at a higher level of professionalism. Now, they could be doing their job. No safety issues. They’re doing what needs to be done. And they could be quite satisfied in their occupation. But we can very easily see the difference between engagement, satisfaction, and how it ties into professionalism. And we had to have tough conversations with not just frontline associates, but even at a leadership level. And we did actually have some leaders early in this journey decide that this wasn’t the ‘bus’ that they wanted to stay on. That’s not easy, right? As you start holding people accountable to what’s expected…that’s a rough road. But we have to be committed to that.” (Medical/Nursing/Other Clinical Interviewee).
	OB5	“So, I think the buzz right now [is] around billing, and being transparent, and people understanding what they’re paying for. …I think, in terms of operating as a business, making sure that our bills are able to be read and understood by patients. They’re itemized, so they understand what went into that charge. And we’ve done a lot of work around that over the last two years, to say: ‘Okay, this is us being transparent and showing them what these billing practices mean.’ So, I think that was a huge step for us in terms of our professionalism to the community.” (Quality/Safety/Performance Improvement/Patient Experience Interviewee).
	OB6	“We have worked very hard to not talk out of one side of our mouth, and then do something opposite. We are trying to be professional in our use of valid, scientific approaches to the management of our company.” (Executive/Operations/Strategy Interviewee).
	OB7	“Organizational professionalism…for me…I would say: the processes and the discipline to process…around how decisions get made, how resources get allocated, how strategy gets set. I think it’s the process around all of those high-level pieces, and the discipline with which the leaders of the organization stick to those processes.” (Executive/Operations/Strategy Interviewee).
	OB8	“Well, I would think it gets back to deployment. Like, how are we executing on our deployment of the Baldrige framework to all of our 40,000 employees. So, getting that deployment all the way to the frontlines. I think that’s probably our most important example of how it manifests itself here daily.” (Executive/Operations/Strategy Interviewee).
*Charter Domains*		
Operations and Business Practices	OB9	“Business practices. I was just writing down ‘just culture,’ because we’ve adopted a just culture approach with our medical staff across the enterprise, with respect to peer review issues. That business practice, I think, has been extremely positive.” (Executive/Operations/Strategy Interviewee).
*Additional Emerging Themes*		
Patient Safety and Experience		See above: PP1, PP2, OC7, CP6, CP10, OB9.
Performance Improvement		See above: OC2, OC7, OC9, CP6, CP7, CP10, CP12, OB3, OB4, OB8, OB9.
Staff Development		See above: OC3, OC4, OC9, CP7, OB3, OB4, OB8.
Core Values		“I think when I hear that ‘organizational professionalism’ idea it’s probably taking these concepts that we talk about all the time – in this case, our organization’s mission, vision, values, of course – but it’s also this role of an anchor institution. And then, how to respond to the changing needs of people.” (Executive/Operations/Strategy Interviewee).
		See above: OC1, OC6, OC11, CP1.
Transparent Communication		See above: CP8, CP11, OB3, OB4, OB5, OB6.
Ethical Leadership		See above: OC8, OC11, CP12, CP13, OB2, OB6, OB9.
*Additional Observations*		
Incorporation of OP Language		See above: OC8, OC10, CP3, CP9, OB7.
Reference to Measurement		See above: PP3, OB1, CP2, CP4, CP6, CP7, CP13.
References to Trust		“I think that systematizing professionalism across an organization allows the organization to be more highly reliable. And the more highly reliable you can be, the more you will gain the trust of the people you are serving.” (Executive/Operations/Strategy Interviewee).
Acknowledgment of OP Principles Interview Shifts Thinking about OP		See above: PP3.
		“I don’t think I’ve ever thought about it before at an organizational level. I guess it’s accepting that there is a set of competencies that the team at the level of the organization must have. It’s not likely that any one individual will have all of them, so…it’s a requirement for people to work together as a team.” (Executive/Operations/Strategy Interviewee).
		“So once I understood where you were trying to go with organizational professionalism, it hasn’t been that hard. But the terminology is not something I was thinking about before this.” (Executive/Operations/Strategy Interviewee).

* Transcripts have been lightly edited for ease of readability.

### Healthcare Leaders’ Reflections on OP Charter Domains and Measurement Tools

The interview then explored the four Charter domains of OP ((1) patient partnerships, (2) organizational culture, (3) community partnerships, and (4) operations and business practices), and asked the interviewee how their organization measured its performance in each domain. Representative quotes from the interviews are presented in **Table 3** and examples of measurement tools are listed in **Table 4**.

**Table 4:** Potential Existing Tools for Measuring OP Identified by Interviewees

Hospital Consumer Assessment of Healthcare Providers and Systems (HCAHPS) Surveys
Other Patient Surveys
Patient Participation in Organization Committees
Patient Engagement Surveys
Community Engagement Surveys
Community Health Needs Assessment
Tracking of Community Volunteers
Tracking of Social Media Presence

#### 1. Patient Partnerships

While the Charter does not rank order the four domains, the concept of patient partnerships was most frequently cited across all interviews. Specifically, leaders often returned to speaking about patient partnerships even when asked about other domains, especially community partnerships. Every leader expressed the belief that there is a strong connection between building trust at the level of individual patients and at the community level (see patient partnership and community partnership quotes in **Table 3**).

We asked interviewees to describe how the organization fosters partnerships with individual patients, such as in making care decisions. Interviewees often used this prompt to speak about how individual- and community-level trust were linked, which initially was surprising since we had anticipated hearing more about topics such as encouraging clinician use of tools to support shared decision-making. The fact that interviewees used this prompt to discuss community relations rather than to talk about decision-making at the individual level may reflect the senior positions these leaders held. These leaders are responsible for their organization’s reputation in the local community in various ways, and they viewed the concept of “patient partnerships” through this lens, while frequently noting that the organization’s relationship with the community depends upon the personal relationships formed between clinicians and patients. Many leaders also described trust flowing in both directions, with patient-level trust in the organization enhanced by efforts to build community trust and vice versa. Specific examples of trust-building activities at the individual patient level were not abundant in interviewee responses, although leaders often described the relationship between community- and individual-level trust, with each benefiting when the other is strengthened. One interviewee stated:

I think that … systematizing professionalism across an organization allows the organization to be more highly reliable. And the more highly reliable you can be, the more you will gain the trust of the people you are serving.

(Executive/Operations/Strategy Interviewee)

When asked about organizational structures to support the quality of patient partnerships, participants most often described patients serving on internal committees, such as Patient and Family Advisory Councils, remarking on how such inclusion can counter a tradition of paternalism in healthcare. One interviewee stated:

It’s not the way that we’ve always thought about providing care, right? It’s always been this top-down approach. “We know better.”

(Medical/Nursing/Other Clinical Interviewee)

In a further reflection of the relationship between community and individual trust, several leaders also referred to building partnerships with community organizations when asked to describe how the organization supports patient partnerships (**Table 3**).

#### 2. Organizational Culture

Interviewees were next asked about organizational culture. To open the conversation, we provided examples of “having a culture of mutual trust among people within the organization” or a “just culture.”

The Baldrige framework includes a set of core values;^12^ given how the interviewees were selected, it is unsurprising that all reported using these values in their performance excellence journey. Interviewees referred to their organization’s core values, often reflecting the Baldrige core values, and described how they were embedded in their organization. For example, one interviewee stated:

I think when I hear that “organizational professionalism” idea, it’s probably taking these concepts that we talk about all the time — in this case, our organization’s mission, vision, values, of course — but it’s also this role of an anchor institution. And then, how to respond to the changing needs of people.

(Executive/Operations/Strategy Interviewee)

Organizational culture was seen as especially related to OP in creating a culture of accountability. Interviewees mentioned ensuring consistent standards, equity, and transparency, and creating an environment where challenging the status quo is encouraged. They frequently spoke about accountability, support, and mutual respect at work. One interviewee stated:

We show up in the morning with our hearts open, and we walk in relationship with each other and with community. We’re responsible for that concept of shared responsibility, relationship, and noticing and valuing dignity in the way that we’re all walking every day.

(Executive/Operations/Strategy Interviewee)

Some healthcare leaders also sought to connect organizational culture to achieving and sustaining public trust, a consistent theme across all of the domains (**Table 3**).

#### 3. Community Partnerships

Interviewees were next asked about their organizations’ relationships to the community, including concepts such as community benefit and engagement, and how they consider or measure professionalism in their community relations. Interviewees acknowledged that community relationships and OP intersect conceptually and practically. They referenced robust organizational collaborations with the communities they serve and other interested parties. Specific strategies mentioned were: appointing senior leaders to be responsible for community relations; being visible in the community; tracking patient experience; establishing patient and family advisory councils (PFACs); working with city, county and state organizations; initiating programs to reduce health disparities; offering scholarships to bring underrepresented minorities into the health professions; and tracking patient and workforce engagement. As noted, community partnerships and patient partnerships were often raised together with similar language. As one interviewee explained:

…The words that I’m using are really challenging the traditional hierarchy in healthcare, where sometimes, as healthcare professionals, we see ourselves as experts. And we think that we know what’s best about how healthcare should be delivered. And, in our minds, we want to break that hierarchy, and instead realize the idea of a partnership where, as healthcare systems … healthcare professionals, we bring our expertise to the table. So, we value what we bring, and equally value what the community brings, and try to put those things together on the table.

(Executive/Operations/Strategy Interviewee)

One unexpected theme that emerged was shared accountability with external organizations. Multiple interviewees spoke about being part of a larger environment than their organization, with examples such as working with city officials to reduce health disparities or working with other organizations on local priorities.

In regard to measurement, the theme of accountability often arose and was seen as related to other themes such as transparency, participation, and being in and of the community, each of which were cited as organizational commitments and responsibilities. Specific measurement tools used included various types of patient engagement surveys and direct patient feedback (both negative and positive), tracking social media traffic, and staff participation levels in community outreach programs such as food drives (**Table 3**).

#### 4. Operations and Business Practices

The fourth domain was fair business practices, which are intended to build relationships of trust with vendors and other business partners as well as with patients. In the Charter, this domain focuses on discussion and decision-making grounded in institutional integrity,^1^ and it encompasses attention to core values while also acknowledging financial and legal considerations.

Most interviewees easily connected the concept of fair business practices and OP; when asked for examples they identified various issues including attention to fair pricing, price transparency, billing practices, and tracking their organization’s impact on the environment. One interviewee stated:

I think the buzz right now around billing and being transparent, and people understanding what they’re paying for in terms of … operating as a business, making sure that our bills are able to be read and understood by patients. They’re itemized, so they understand what went into that charge. And we’ve done a lot of work around that over the last two years, to say: “Okay, this is us being transparent and showing them what these billing practices mean.” So, I think that was a huge step for us in terms of our professionalism to the community.

(Quality/Safety/Performance Improvement/Patient Experience Interviewee)

When asked about ways to measure fair business practices, several leaders cited corporate compliance reporting as a key means of tracking organizational performance in this domain. They also identified comparative benchmarking as a way to ensure their organizations were not outliers in the marketplace. Of note, the Baldrige assessment requires that organizations identify relevant comparators and benchmark their performance on multiple metrics over time against these benchmark organizations as well as industry standards. Since the Baldrige assessment is data-driven, all interviewees reported extensively documenting performance and use of sophisticated, organization-wide data systems.

Interviewees also often identified specific examples of interactions in the community as measures of their organization’s fair business practices, as well as their organization’s role in the community as employer, revenue generator, and promoter of diversity, equity, and inclusion. These examples suggest a potential overlap and opportunity for coordination with organizational activities in the community partnership domain **(Table 3)**.

### Additional Themes Identified

Beyond the four proposed domains, six additional themes related to OP emerged: (1) patient safety and experience; (2) performance improvement; (3) staff development; (4) organizational core values; (5) clear and transparent communication; and (6) ethical leadership. While all of these might be considered to fall under the “Organizational Culture” domain of OP, their identification as distinct themes by these leaders points to the need for further work on how to describe and define OP in ways useful to healthcare delivery organizations.

## Discussion

Very few healthcare executives we interviewed had heard the term “organizational professionalism” prior to being interviewed, yet all readily connected their operating processes and strategic decisions to it and could readily summon examples of how their organization attended to all four of the domains of OP, including ways in which they measured performance in each domain. This suggests that principles of OP may be embedded within the leadership and culture of these high-performing healthcare organizations, albeit with variations in how the concept is described and where related principles and practices are applied and measured. It is also remarkable that all leaders interviewed readily adopted the term during interviews, and several reported that further exploration of this concept could be useful to them as leaders developing cross-cutting initiatives around OP concepts.

We were especially interested in how organizations measured performance in each of the four domains of OP (patient partnerships, organizational culture, relationships with the community, and fair business practices). We heard a number of potentially useful ideas for each. In general, reported organizational measurement and tracking strategies included using surveys, various community engagement metrics, organizational business and compliance metrics, and in-person observations to identify and reward employee actions that directly exemplify professionalism. The fact that these measures already exist and are being used raises the potential for creating an OP “dashboard,” with a common set of recommended metrics for tracking (**Table 4**).

The additional themes offered by these leaders, which were sometimes seen as distinct from the four Charter domains, suggest that the concept of OP still needs greater clarity or breadth, or both, to fully encompass the ways in which healthcare leaders interpret, implement, and measure OP within their organizations **(Table 3)**. At the same time, the frequent references to trust and its relationship to OP across all four domains also suggest a potential unifying strategy for promoting attention to OP by focusing on its presumed impacts on patient and community trust in healthcare systems.

## Limitations and Future Research

While this study is notable for being the first formal exploration of the concept of OP among health system leaders, it has several limitations, and it provides important fodder for future research. First, there is a preexisting definition of OP which we adopted, but the utility of this definition was, itself, a subject for this study. Examining a concept that does not yet have a well-accepted definition creates obvious challenges, which is why we used qualitative methods for this initial investigation. Future work should seek to further define OP, including consideration of the additional themes raised by these leaders and realistic measurement strategies for each proposed domain.

Second, there is no list of organizations that have sought to or do excel in OP, so we used the proxy measure of being a recent recipient of a Baldridge award. The Baldridge award examines concepts that are intuitively similar to those in the OP definition we used **(Table 2)**, but they are not the same. It is likely that we found more similarities between Baldridge language and OP language and concepts than we might have if we had not selected these organizations. Future work might benefit from examining views of leaders in organizations that use other frameworks for performance improvement, such as Anchor Institution status^13^ or Lean management.^14^

Third, this study was descriptive, not normative. We explored how current leaders think about and use the concept of OP, but not what they would recommend for themselves or others. This will be especially important for future work on developing an OP measurement dashboard, and future research should carefully examine any proposed set of metrics for their relationship to important outcomes such as clinical outcomes or patient and community trust.

## Conclusion

Enhancing the understanding of OP in healthcare organizations has the potential to establish and sustain professional and ethical organizational cultures that bolster trust through the sound implementation of laws, policies, and procedures to support the delivery of high-quality patient care. We therefore undertook this qualitative study to explore how the concept of “organizational professionalism,” as described in the OP Charter, is understood and potentially measured by leaders of high-performing healthcare organizations. The four OP domains identified in the Charter guided our interviews and data analysis, but we found very few leaders were already familiar with the concept of OP. Nonetheless, the concept resonated as important to their organizations, and they easily provided various examples of how their organizations attended to and measured performance in each of the four OP domains. These examples arose in myriad organizational contexts, which is not surprising since there is, at present, no overall set of policies and practices that fall under the rubric of OP. Further, our findings suggest both conceptual overlap across Charter domains — at least from the point of view of organizational leaders, who often returned conversations to the goal of enhancing community trust — as well as some new areas that might be included as aspects of OP. This is a formative descriptive study, which raises more questions than it answers. Many future opportunities exist to better define and align Charter domains to enhance the practical application of OP concepts in healthcare delivery organizations.

## Note

The authors are indebted to the senior leaders of the eight study organizations who generously gave their time for interviews. The authors also appreciate the advice provided by leaders of the Baldrige Performance Excellence Program. The authors offer their gratitude to the Professionalism Grant Project Group Executive Committee (PGPG EC) members for their invaluable guidance, direction, and support throughout this project. Members of the Professionalism Grant Project Group (PGPG) Executive Committee (EC) include: Julie L. Agris, Ph.D., J.D., LL.M., FACHE (Chair) of the Shepard Broad College of Law, Nova Southeastern University; Barry E. Egener, M.D., of Asante Health; Dennis H. Novack, M.D. of Drexel University College of Medicine; Sherril B. Gelmon, Dr.P.H., of the OHSU-PSU School of Public Health; Matthew Wynia, M.D., M.P.H., of the University of Colorado, Anschutz Medical Campus; Richard M. Frankel, Ph.D., of the Indiana University School of Medicine; William Nelson, Ph.D., M.Div., of the Geisel School of Medicine at Dartmouth College; and Fred Hafferty, Ph.D., of the Mayo Clinic.
